# The Phytochemistry, Pharmacology, and Quality Control of *Tetrastigma hemsleyanum* Diels & Gilg in China: A Review

**DOI:** 10.3389/fphar.2020.550497

**Published:** 2020-09-25

**Authors:** Ruyi Zhu, Xiaofen Xu, Jialiang Ying, Gang Cao, Xin Wu

**Affiliations:** College of Pharmaceutical Sciences, Zhejiang Chinese Medical University, Hangzhou, China

**Keywords:** *****Tetrastigma hemsleyanum* Diels et Gilg, phytochemistry, total flavonoids, pharmacological activities, quality control

## Abstract

*Tetrastigma hemsleyanum* Diels & Gilg (TDG), the family member of Vitaceae, is a traditional herbal medicine in China. The root of TDG can be immediately used after cleaning the muddy soil, and can be dehydrated for dry use. TDG is able to be collected all year round, which is commonly used in the treatment of hepatitis, infantile high fever, snake bite, etc. Based on phytochemistry, the chemical components of TDG are divided into flavonoids, phenolic acids, terpenes, steroids, polysaccharide, and other compounds, showing many pharmacological effects which include anti-tumor, anti-oxidation, anti-inflammatory, antipyretic, analgesic, and immunomodulatory activity, as well as other activities. Currently, TDG involves some problems of the reduction of wild resources, the backward processing methods, and storage difficulties as well as the imperfection of detection methods. Therefore, this review summarizes the literature of the past 20 years, and the purpose of this review is to summarize the recent researches on the phytochemistry, pharmacology, quality control, and clinical application of TDG. The above discussions provide new insights for the future research on TDG.

## Introduction

*Tetrastigma hemsleyanum* Diels & Gilg (abbreviated as TDG) is a perennial Liana plant of family Vitaceae. Firstly recorded in Textual Research on Reality and Titles of Plants (Wu, 1957), it is successively recorded in various medical books and literatures. TDG is widely used as a traditional medicine with its root being used after washing or processing (Zhejiang Food and Drug Administration, 2015; Ding F. et al., 2018; Ding Z. et al., 2018). It is mainly distributed in the south regions along the Yangtze River, such as Zhejiang province, Fujian province, Guangxi province, and so on. TDG is bitter, pungent, and cool in nature. Then, the tuberous roots or whole grass of TDG can be commonly used as medicine, with the effect of clearing away heat and detoxification, eliminating swelling and pain, promoting blood circulation and removing blood stasis, dispelling wind and phlegm. In the clinical practice of traditional Chinese medicine, TDG, which can be internally or externally taken, is generally used to treating children’s febrile convulsions, hepatitis, snake bites, cellulitis, and other diseases (Ji et al., 2014). The results show that TDG is abundant in plant chemical components and a variety of biological activities, while with unclear amount of specific active substances. Moreover, according to modern pharmacology studies, TDG has many pharmacological effects, including anti-tumor, anti-inflammatory, anti-oxidation, antipyretic and analgesic, and liver protection. It can be used as the main elements for many kinds of Chinese patent drugs and health products, such as Huatuofengtongbao capsule, Paishilidan capsule, Jieshikang capsule, Jinsidijia capsule, Jinqi tablet, etc. (Peng et al., 2016a).

**Figure 1:**
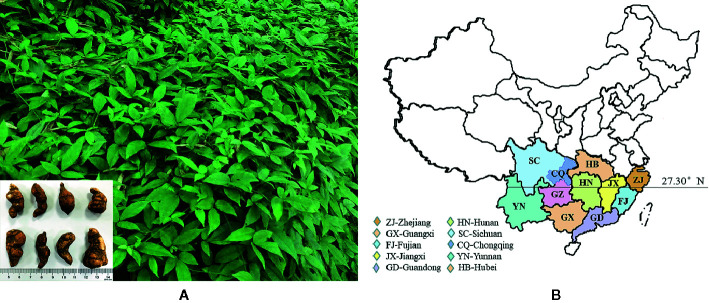
The aerial stem leaf and underground tuber of *Tetrastigma hemsleyanum* Diels & Gilg in Zhejiang **(A)**; Distribution map of wild resources of *Tetrastigma hemsleyanum* Diels & Gilg **(B)**.

*Tetrastigma hemsleyanum* Diels & Gilg (it’s called Sanyeqing in China, **Figure 1A**) is a perennial grass climbing vine (Ye, 2011). TDG is basically divided into purple rattan TDG and green rattan TDG. The former is purple brown near the root, while the latter is in blue white and does not grow in combination with the dimension of 27.3^°^ in China (Fan et al., 2018) (as shown in **Figure 1B**). In contrast, green rattan TDG is widely distributed with a higher yield, while the purple rattan TDG has the better medicinal value. Its section is white, one or more connected, beaded. The best harvest period is the winter solstice (Liu and Wei, 2018). Wild species often grow in the shade of valley forest and cliff, mostly scattered in Zhejiang province, Fujian province, Guangxi province, Guangdong province, Jiangxi province, Sichuan province, Chongqing province, Hunan province, Guizhou province, and other provinces, sensitive to cold climate. When the temperature drops to 10°C in winter, the growth will be stagnant and appears to be drought resistant and cannot accumulate water (Liu and Wei, 2018). The stem and branches are slender, longitudinally ribbed, glabrous, or sparsely pilose. Compound leaves are palmate, usually foliolating with three blades. There are spiny sparse teeth on the edge. Flowers are small in the color between yellow and green, inflorescence axillary, as well as being closed like umbrellas. The fruit is red globose when it ripens. The tuber root is spindle-shaped, elliptical, or oval, and with tan brown but smooth surface. Its section is white, single or multiple, and connected with each other, presenting bead shape (Editorial Board of “Chinese Materia Medica” of the State Administration of Traditional Chinese Medicine, 1999). The microscopic characteristics show that the vessels are mostly marginal pits in addition to rare calcium oxalate cluster crystals existed or scattered in mucus cells. Moreover, some contain calcium oxalate needle crystals with bundles or scattered forms. Besides, the slender fibers exist in single or bundles with many oval and quasi round starch grains. Furthermore, different producing areas may affect the appearance, size, and microscopic characters of TDG. For instance, the tubers of TDG from Zhejiang province are small with a smooth surface and the white color of cross-section; while the tubers from Guangxi are larger, with wrinkled epidermis and pink color of cross-section. In addition, the vascular bundles of TDG from Guangxi are arranged in a radial pattern, while those in Zhejiang province are arranged in the shape of “> <“ (Huang et al., 2007; Yu et al., 2018; Cui et al., 2019). The best harvest time is the winter solstice when its underground root tuber plays the best medicinal effect (Liu and Wei, 2018).

In recent years, due to the increasing wild resources of TDG have been over exploited, resulting in a phenomenon of mixed use of drugs with similar appearance and similar efficacy on the market. Therefore, on the basis of reviewing relevant literature at home and abroad, this paper systematically summarizes the botany, phytochemistry, pharmacological activities, quality control, and clinical application of TDG. The aim is to provide valuable reference for the future development and the application of TDG.

## Phytochemistry

Nowadays, a great deal of studies on the chemical constituents of TDG have been performed. The results show that there are flavonoids, phenolic acids, fatty acids, triterpenoids, steroids, and other compounds in TDG, especially flavonoids and phenolic acids. Among them, flavonoids are the most valuable active components isolated from the herb, and have significant anti-tumor effects.

### Flavonoids

**Figure 2:**
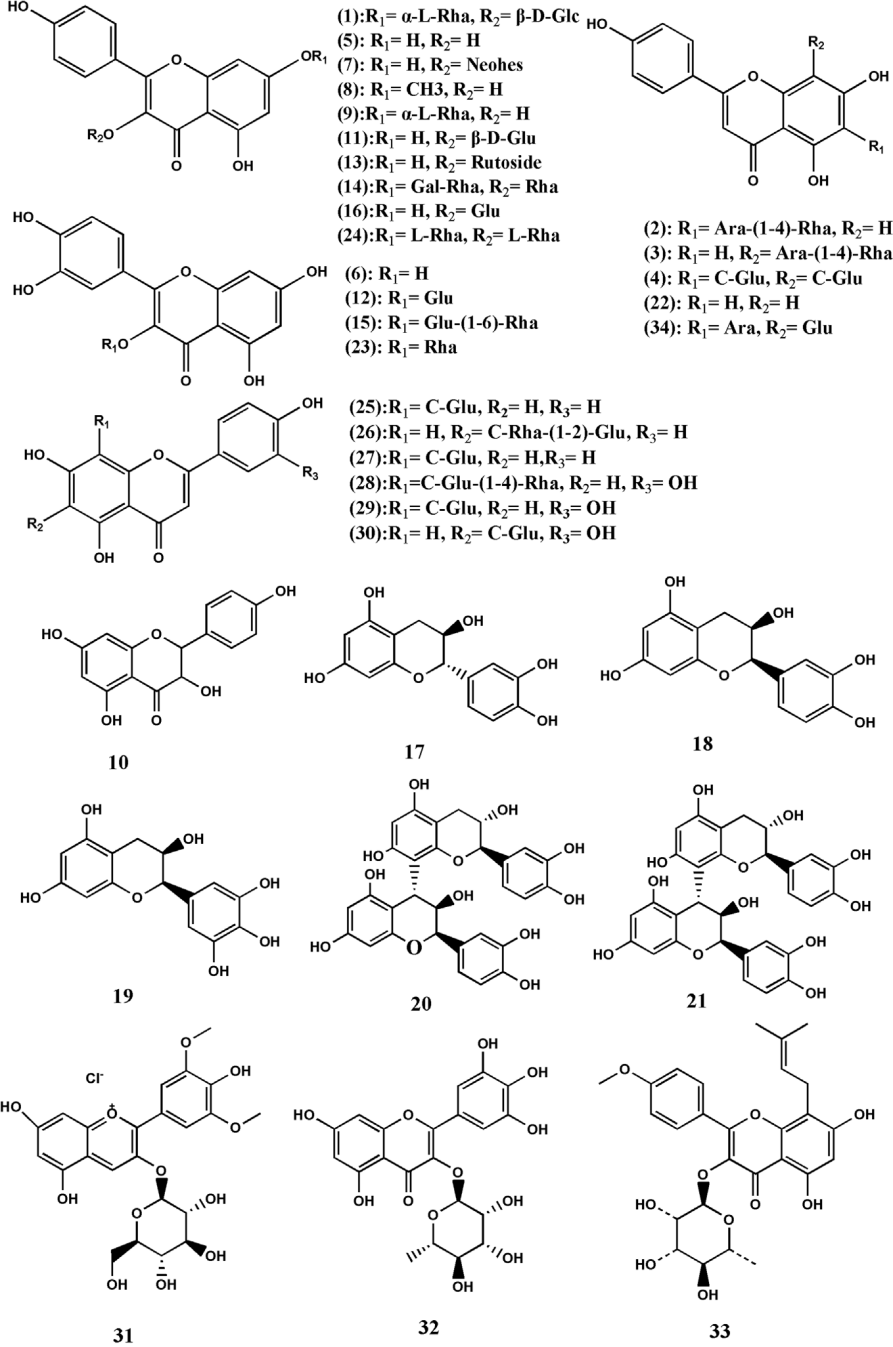
Structures of the flavonoids from *Tetrastigma hemsleyanum* Diels & Gilg.

Flavonoids and their glycosides are considered to be one of the most abundant chemical constituents of TDG, with a lot of research results. At present, most of the studies are mainly focused on the roots of the medicinal part of TDG, while the studies on the aerial part are less. At the same time, some studies have found that the aerial part of TDG contains many active substances, which have functions such as anti-tumor, anti-bacterial, anti-inflammatory, and so on. In addition, some researchers also measure the total flavone content of different batches of TDG, and they determine the total flavone content in the range of 0.18–0.66% (Yu et al., 2018). In addition, the study has found that the content of total flavonoids in leaves of TDG ranges from 13.38 to 28.67 mg· g^-1^ (Fan et al., 2017). More than 30 flavonoids isolated and identified from TDG, including kaempferol-7-O-α-L-rhamnopyranosyl-3-O-β-D-glucopyranoside (1), apigenin-6-C-α-L-arabinopyranosyl-(1-4)-α-L-rhamnopyranoside (2), and apigenin-8-C-α-L-arabinopyranosyl-(1-4)-α-L-rhamnopyranoside (3), apigenin-6, 8-di-C-β-D-glucopyranoside (4), kaempferol (5), quercetin (6), kaempferol 3-neohesperidoside (7), rhamnocitrin (8), kaempferol-7-O-α-L-rhamnopyranoside (9), aromadendrin (10), kaempferol-3-O-β-D-glucopyranoside (11), isoquercetin (12), nicotifiorin (kaempferol-3-o-rutoside, 13), robinin (14), rutin (15), astragaloside (16), catechin (17), L-epicatechin (18), epigallocatechin (19), procyanidin B2 (20), procyanidin B1 (21), apigenin (22), quercitrin (23), kaempferitrin (24), apigenin-6-C-β-D-glucopyranoside (Isovitexin, 25), apigenin-8-C-α-L-rhamnopyranosyl-(1-2)-β-D-glucopyranoside (Vitexin-2-O-rhamnoside, 26), apigenin-8-C-β-D-glucopyranoside (Vitexin, 27), apigenin-8-C-β-D-glucopyranoside-(1-4)-β-D-glucopyranoside (Vitexin-4’’-O-glucoside, 28), orientin (29), isoorientin (30), malvidin-3-glucoside (31), myricitrin (32), baohuosid I (33), and isoschaftoside (34) (Liu, 2000; Liu et al., 2002; Li et al., 2003; Guo, 2013; Zeng, 2013; Fan et al., 2014; Fu et al., 2015; Lin et al., 2015; Liu et al., 2018). The structure of some flavonoids is shown in **Figure 2**, with the names of all constituents being listed in **Table 1**.

**Table 1:** Flavonoids isolated from *Tetrastigma hemsleyanum* Diels & Gilg.

No.	Name	Formula	Molecular Weight	[M-H]^-^ or [M+H]^+^ (m/z)	MS/MS fragments (m/z)	References
**1**	Kaempferol-7-O-α-L-rhamnopyranosyl-3-O-β-D-glucopyranoside	C_27_H_30_O_15_	–	595 (+)	286, 258, 121	(Liu, 2000)
**2**	Apigenin-6-C-α-L-arabinopyranosyl-(1-4)-α-L-rhamnopyranoside	C_26_H_28_O_13_	–	549 (+)	384 (30), 283(100), 270 (61), 165 (30)	(Liu et al., 2002)
**3**	Apigenin-8-C-α-L-arabinopyranosyl-(1-4)-α-L-rhamnopyranoside	C_26_H_28_O_13_	–	549 (+)	384 (30), 283(100), 270 (61), 165 (30)	(Liu et al., 2002)
**4**	Apigenin-6,8-di-C-β-D-glucopyranoside	C_27_H_30_O_15_	–	595 (+)	384 (30), 283(100), 270 (61), 165 (30)	(Liu et al., 2002)
**5**	Kaempferol	C_15_H_10_O_6_	–	–	–	(Li et al., 2003)
**6**	Quercetin	C_15_H_10_O_7_	–	–	–	(Li et al., 2003)
**7**	Kaempferol 3-Neohesperidoside	C_27_H_30_O_15_	–	594. 9 (−)	448.8, 287.1	(Li et al., 2003)
**8**	Rhamnocitrin	C_16_H_12_O_6_	–	301 (+)	–	(Zeng, 2013)
**9**	Kaempferol-7-O-α-L-rhamnopyranoside	C_21_H_20_O_10_	–	433(+)\431(−)	–	(Zeng, 2013)
**10**	Aromadendrin	C_15_H_12_O_6_	–	287 (+)	–	(Guo, 2013)
**11**	Kaempferol-3-O-β-D-glucopyranoside	C_21_H_20_O_11_	–	–	–	(Guo, 2013)
**12**	Isoquercetin	C_21_H_20_O_12_	–	–	–	(Guo, 2013)
**13**	Nicotifiorin (kaempferol-3-o-rutoside)	C_27_H_30_O_15_	–	593 (−)	593,447,285	(Guo, 2013)
**14**	Robinin	C_33_H_40_O_19_	–	739 (−)	–	(Guo, 2013)
**15**	Rutin	C_27_H_30_O_16_	–	609.1460 (−)	–	(Fan et al., 2014)
**16**	Astragalin	C_21_H_20_O_11_	–	447.0922 (−)	–	(Fan et al., 2014)
**17**	Catechin	C_15_H_14_O_6_	–	289.0712 (−)	–	(Fu et al., 2015)
**18**	L-Epicatechin	C_15_H_14_O_6_	–	289.0710 (−)	–	(Fu et al., 2015)
**19**	Epigallocatechin	C_15_H_14_O_7_	–	305.0655 (−)	–	(Fu et al., 2015)
**20**	Procyanidin B2	C_30_H_26_O_12_	–	577.1348 (−)	–	(Fu et al., 2015)
**21**	Procyanidin B1	C_30_H_26_O_12_	–	577.1344 (−)	–	(Fu et al., 2015)
**22**	Apigenin	C_15_H_10_O_5_	–	431.0972 (−)	–	(Lin et al., 2015)
**23**	Quercitrin	C_21_H_20_O_11_	–	447.0925 (−)	–	(Lin et al., 2015)
**24**	Kaempferitrin	C_27_H_30_O_14_	–	577.1554 (−)	–	(Lin et al., 2015)
**25**	Apigenin-6-C-β-D-glucopyranoside	C_21_H_20_O_10_	–	431.0978 (−)	–	(Lin et al., 2015)
**26**	Apigenin-8-C-α-L-rhamnopyranosyl-(1-2)-β-D-glucopyranoside	C_27_H_30_O_14_	–	577.1558 (−)	–	(Lin et al., 2015)
**27**	Apigenin-8-C-β-D-glucopyranoside	C_21_H_20_O_10_	–	431.0972 (−)	–	(Lin et al., 2015)
**28**	Apigenin-8-C-β-D-glucopyranoside-(1-4)-β-D-glucopyranoside	C_27_H_30_O_15_	–	593.1506 (−)	–	(Lin et al., 2015)
**29**	Orientin	C_21_H_20_O_11_	–	447.0928 (−)	–	(Lin et al., 2015)
**30**	Isoorientin	C_21_H_20_O_11_	–	447.0929 (−)	–	(Lin et al., 2015)
**31**	Malvidin-3-glucoside	C_23_H_25_ClO_12_	528.92	–	–	(Liu et al., 2018)
**32**	Myricitrin	C_21_H_20_O_12_	464.38	–	–	(Liu et al., 2018)
**33**	Baohuosid I	C_27_H_30_O_10_	514.53	–	–	(Liu et al., 2018)
**34**	Isoschaftoside	C_26_H_28_O_14_	564.94	–	–	(Liu et al., 2018)

“-”: Some relevant information in the literature is incomplete.

(−): [M-H] ^-^ (m/z).

(+): [M+H] ^+^ (m/z).

### Phenolic Acids

**Figure 3:**
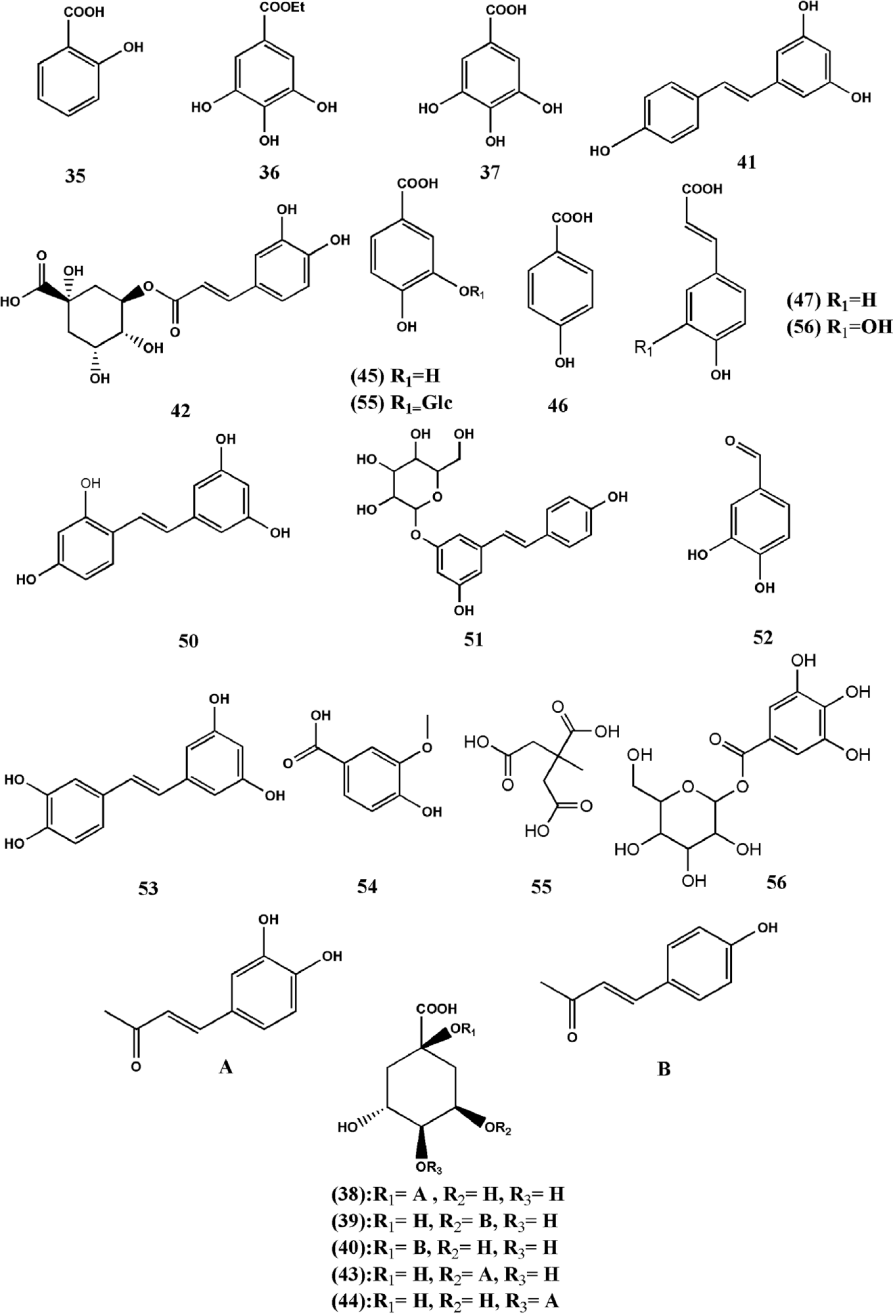
Structures of phenolic acids from *Tetrastigma hemsleyanum* Diels & Gilg.

Phenolic acids are considered as the important plant compounds in TDG, possessing excellent anti-inflammatory, anti-oxidation, and anti-tumor activities. At present, there are more than 30 phenolic acids, mainly including salicylic acid (35) and ethyl gallate (36), gallic acid (37), 1-caffeoylquinic acid (38), 5-p-coumaroylquinic acid (39), 1-p-coumaroylquinic acid (40), resveratrol (41), chlorogenic acid(42), neochlorogenic acid (43), cryptochlorogenic acid (44), 3,4-Dihydroxybenzoic acid (45), 4-hydroxybenzoic acid (46), trans-4-hydroxycinnamic acid (47), oxyresveratrol (48), protocatechualdehyde (49), piceatannol (50), polydatin(51), vanillic acid (52), citric acid (53), glucosamine (54), protocatecheuic acid 3-O-β-D-glucoside (55), caffeic acid (56) (Liu, 2000; Sun et al., 2013; Zeng, 2013; Chen, 2014; Xu et al., 2014a; Ding et al., 2015b; Fu et al., 2015; Jiang, 2015; Lin et al., 2016; Sun C. et al., 2018). The chemical structures of above compounds are shown in **Figure 3**, in the names of all the constituents being listed in **Table 2**.

**Table 2:** Phenolic acids isolated from *Tetrastigma hemsleyanum* Diels & Gilg.

No.	Name	Formula	Molecular Weight	[M-H]^-^ or [M+H]^+^ (m/z)	MS/MS fragments (m/z)	References
**35**	Salicylic acid	C_7_H_6_O_3_	–	–	138 (60), 120 (100), 92 (72)	(Liu, 2000)
**36**	Ethyl gallate	C_9_H_10_O_5_	198	–	198(52), 170 (20), 153 (100), 125(18)	(Liu, 2000)
**37**	Gallic acid	C_7_H_6_O_5_	170	–	170 (100), 152 (90), 135(22), 125(30), 107(16), 79(28)	(Liu, 2000)
**38**	1-Caffeoylquinic acid	C_16_H_18_O_9_	–	353.0878 (−)	191.0569, 127.0196	(Sun et al., 2013)
**39**	5-p-Coumaroylquinic acid	C_16_H_18_O_8_	–	337.0929 (−)	191.0565, 163.0425	(Sun et al., 2013)
**40**	1-p-Coumaroylquinic acid	C_16_H_18_O_8_	–	337.0929 (−)	191.0568	(Sun et al., 2013)
**41**	Resveratrol	C_14_H_12_O_3_	–	227 (−)	–	(Zeng, 2013)
**42**	Chlorogenic acid	C_16_H_18_O_9_	–	353. 0874 (−)	191	(Xu et al., 2014a)
**43**	Neochlorogenic acid	C_16_H_18_O_9_	–	353. 0887 (−)	191	(Xu et al., 2014a)
**44**	Cryptochlorogenic acid	C_16_H_18_O_9_	–	353. 0883 (−)	191	(Xu et al., 2014a)
**45**	3,4-Dihydroxybenzoic acid	C_7_H_6_O_4_	–	155.16 (+)	–	(Chen, 2014)
**46**	4-Hydroxybenzoic acid	C_7_H_6_O_3_	–	139.16 (+)	–	(Chen, 2014)
**47**	Trans-4-hydroxycinnamic acid	C_9_H_8_O_3_	–	165.15 (+)	–	(Chen, 2014)
**48**	Oxyresveratrol	C_14_H_12_O_4_	–	243.0651 (−)	–	(Fu et al., 2015)
**49**	Protocatechualdehyde	C_7_H_6_O_3_	–	137.0237 (−)	–	(Fu et al., 2015)
**50**	Piceatannol	C_14_H_12_O_4_	–	–	–	(Jiang, 2015)
**51**	Polydatin	C_20_H_22_O_8_	–	–	–	(Jiang, 2015)
**52**	Vanillic acid	C_8_H_8_O_4_	168	–	168	(Ding et al., 2015b)
**53**	Citric acid	C6H8O7	–	191.0183 (−)	147, 112	(Sun C. et al., 2018)
**54**	Gallic acid hexoside	C_13_H_16_O_10_	–	331.0643 (−)	169, 125	(Sun C. et al., 2018)
**55**	Protocatecheuic acid 3-O-β-D-glucoside	C_13_H_16_O_9_	–	315.0523 (−)	153, 152, 109, 108	(Sun C. et al., 2018)
**56**	Caffeic acid	C_9_H_8_O_4_	–	179.0342 (−)	135	(Sun C. et al., 2018)

“-”: Some relevant information in the literature is incomplete.

(−): [M-H] ^-^ (m/z).

(+): [M+H] ^+^ (m/z).

### Triterpenoids and Steroids

**Figure 4:**
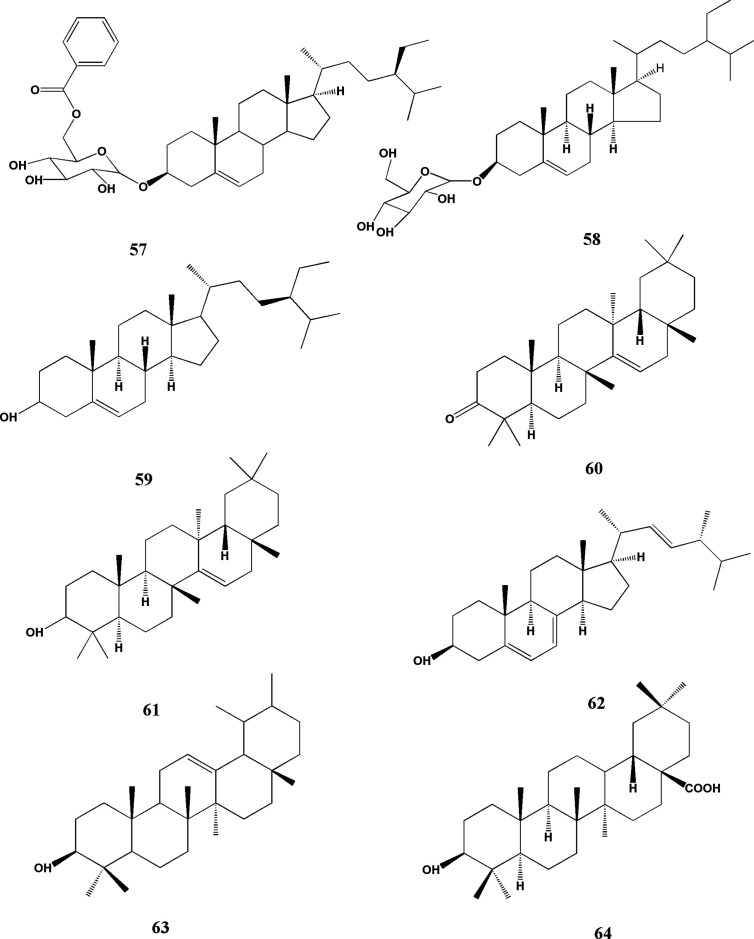
Structures of triterpenoids and steroids from *Tetrastigma hemsleyanum* Diels & Gilg.

Triterpenes and steroids are common secondary metabolites in plants. Now, nine compounds with clear structure have been isolated from TDG. The major compounds include 6’-O-benzoyldaucosterol (57), daucosterol (58), β-sitosterol (59), taraxerone (60), taraxerol (61), ergosterol (62), α-amyrin (63), oleanolic acid (64) (Yang et al., 1998; Liu and Yang, 1999; Liu, 2000; Ding et al., 2015b), which are shown in **Figure 4** in the name of eight compounds listed in **Table 3**. It is found that β-sitosterol exists in different extracts of TDG and may be one of its active components, which needs to be further studied.

**Table 3:** Triterpenoids and steroids isolated from *Tetrastigma hemsleyanum* Diels & Gilg.

No.	Name	Formula	Molecular Weight	[M-H]^-^ or [M+H]^+^ (m/z)	MS/MS fragments (m/z)	References
**57**	6′-O-Benzoyldaucosterol	C_42_H_64_O_7_	–	–	414, 396, 163, 121, 105	(Yang et al., 1998)
**58**	Daucosterol	C_35_H_60_O_6_	–	–	414, 396, 163	(Yang et al., 1998)
**59**	β-Sitosterol	C_29_H_50_O	–	–	414, 396, 381	(Yang et al., 1998)
**60**	Taraxerone	C_30_H_48_O	424	–	424 (30), 409 (30), 300 (90), 285 (70), 272 (20), 257 (19), 205 (55), 204 (100), 189 (30), 133 (85), 121 (45)	(Liu and Yang, 1999)
**61**	Taraxerol	C_30_H_50_O	426	–	426 (30),411 (40), 393(10), 302 (48), 287 (40), 269 (15),218 (20), 204 (100), 189 (25), 135 (70)	(Liu and Yang, 1999)
**62**	Ergosterol	C_28_H_44_O	396	–	396(100), 381 (2), 363 (63), 271 (13), 269 (5), 253 (30)	(Liu and Yang, 1999)
**63**	α-Amyrin	C_30_H_50_O	426	–	426 (50), 411 (46), 393 (40), 302 (32), 287 (29), 269 (20), 218 (30), 204 (100), 189 (30), 135 (70)	(Liu, 2000)
**64**	Oleanolic acid	C_30_H_48_O_3_	456	–	–	(Ding et al., 2015b)

“-”: Some relevant information in the literature is incomplete.

(−): [M-H] ^-^ (m/z).

(+): [M+H] ^+^ (m/z).

### Volatile Oil and Fatty Acids

**Figure 5:**
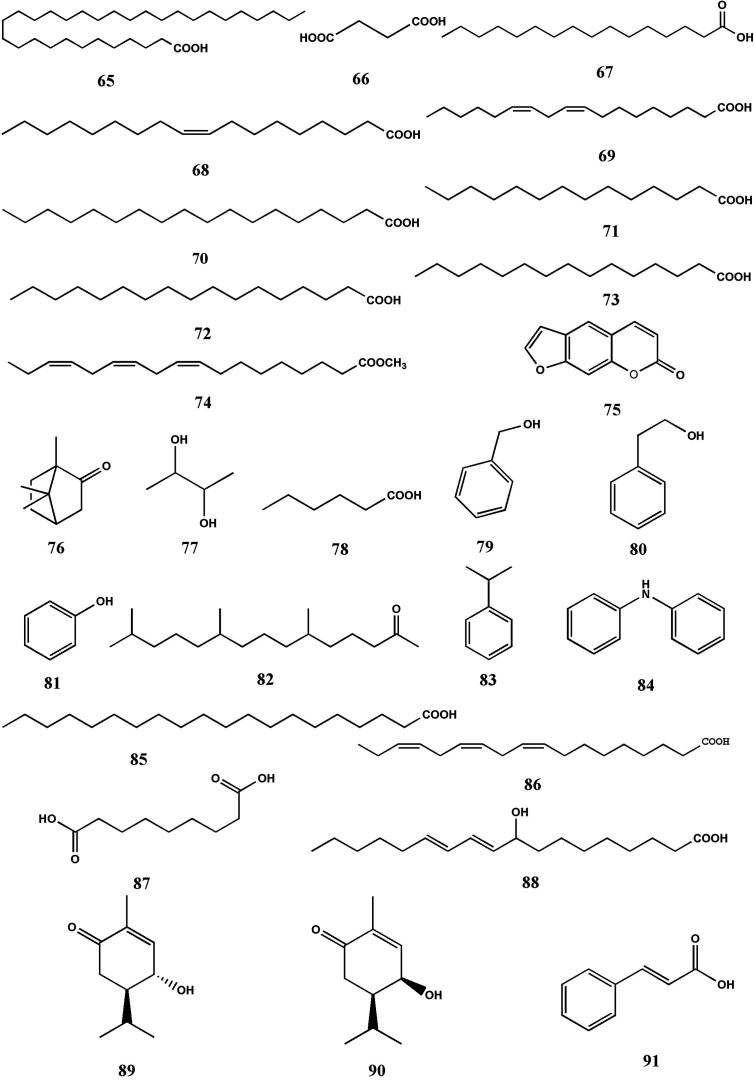
Structures of volatile oil and fatty acids from *Tetrastigma hemsleyanum* Diels & Gilg.

Fatty acids are part and parcel in TDG. Nowadays, a variety of fatty acids and volatile oils have been identified in TDG, mainly including lacceroic acid (65), succinic acid (66), palmitic acid (67), oleic acid (68), linoleic acid (69), stearic acid (70), myristic acid (71), margaric acid (72), pentadecylic acid (73) and methyl linolenate (74), psoralen (75), camphor (76), 2, 3-butanediol (77), hexanoic acid (78), benzyl alcohol (79), benzeneethanol (80), phenol (81), 6, 10, 14-trimethyl-2-pentadecanone (82), cumene (83), diphenylamine (84), arachidic acid (85), linolenic acid (86), azelaic acid (87) (Liu, 2000; Huo et al., 2008; Hu et al., 2013; Ding et al., 2015b). Xu et al. (2017) isolate a long-chain polyene unsaturated fatty acid component, three kinds of monoterpenes and one kind of phenylpropanoids from the extraction site of petroleum ether, which are 9-hydroxy-10,12-octadecadienoic acid (88), (4R, 5R)-4-Hydroxy-2-methyl-5-propan-2-ylcyclohex-2-en-1-one (89), (4S, 5R)-4-Hydroxy-2-methyl-5-propan-2-ylcyclohex-2-en-1-one (90), (3R, 4R, 6S)-3, 6-dihydroxy-1-menthylene, cinnamic acid (91), respectively. The chemical structures of volatile oil and fatty acids are shown in **Figure 5**, in the name of the compounds listed in **Table 4**.

**Table 4:** Volatile oil and fatty acids from *Tetrastigma hemsleyanum* Diels & Gilg.

No.	Name	Formula	Molecular Weight	[M-H]^-^ or [M+H]^+^ (m/z)	MS/MS fragments (m/z)	References
**65**	Lacceroic acid	C_32_H_64_O_2_	480	–	480 (20), 129 (38), 111 (17), 85 (37), 73 (65),57 (100)	(Liu, 2000)
**66**	Succinic acid	C_4_H_6_O_4_	–	–	119 (60),101 (100)	(Liu, 2000)
**67**	Palmitic acid	C_16_H_32_O_2_	256	–	–	(Huo et al., 2008)
**68**	Oleic acid	C_18_H_34_O_2_	282	–	–	(Huo et al., 2008)
**69**	Linoleic acid	C_18_H_32_O_2_	280	–	–	(Huo et al., 2008)
**70**	Stearic acid	C_18_H_36_O_2_	284	–	–	(Huo et al., 2008)
**71**	Myristic acid	C_14_H_28_O_2_	228	–	–	(Huo et al., 2008)
**72**	Margaric acid	C_17_H_34_O_2_	270	–	–	(Huo et al., 2008)
**73**	Pentadecylic acid	C_15_H_30_O_2_	242	–	–	(Huo et al., 2008)
**74**	Methyl linolenate	C_19_H_32_O_2_	292	–	–	(Huo et al., 2008)
**75**	Psoralen	C_11_H_6_O_3_	186	–	–	(Huo et al., 2008)
**76**	Camphor	C_10_H_16_O	152	–	–	(Huo et al., 2008)
**77**	2, 3-Butanediol	C_4_H_10_O_2_	90	–	–	(Huo et al., 2008)
**78**	Hexanoic acid	C_6_H_12_O_2_	116	–	–	(Huo et al., 2008)
**79**	Benzyl alcohol	C_7_H_8_O	108	–	–	(Huo et al., 2008)
**80**	Benzeneethanol	C_8_H_10_O	122	–	–	(Huo et al., 2008)
**81**	Phenol	C_6_H_6_O	94	–	–	(Huo et al., 2008)
**82**	6,10,14-trimethyl-2-pentadecanone	C_18_H_36_O	268	–	–	(Huo et al., 2008)
**83**	Cumene	C_9_H_12_	120	–	–	(Huo et al., 2008)
**84**	Diphenylamine	C_12_H_11_N	169	–	–	(Huo et al., 2008)
**85**	Arachidic acid	C_20_H_40_O_2_	312	–	–	(Hu et al., 2013)
**86**	α-Linolenic acid	C_18_H_30_O_2_	278	–	–	(Hu et al., 2013)
**87**	Azelaic acid	C_9_H_16_O_4_	–	–	–	(Ding et al., 2015b)
**88**	9-hydroxy-10,12-octadecadienoic acid	C_18_H_32_O_3_	–	297 (+)	–	(Xu et al., 2017)
**89**	(4R, 5R)-4-Hydroxy-2-methyl-5-propan-2-ylcyclohex-2-en-1-one	C_10_H_16_O_2_	–	169 (+)	–	(Xu et al., 2017)
**90**	(4S, 5R)-4-Hydroxy-2-methyl-5-propan-2-ylcyclohex-2-en-1-one	C_10_H_16_O_2_	–	169 (+)	–	(Xu et al., 2017)
**91**	Cinnamic acid	C_9_H_8_O_2_	–	149 (+)	–	(Xu et al., 2017)

“-”: Some relevant information in the literature is incomplete.

(−): [M-H] ^-^ (m/z).

(+): [M+H] ^+^ (m/z).

### The Other Components

**Figure 6:**
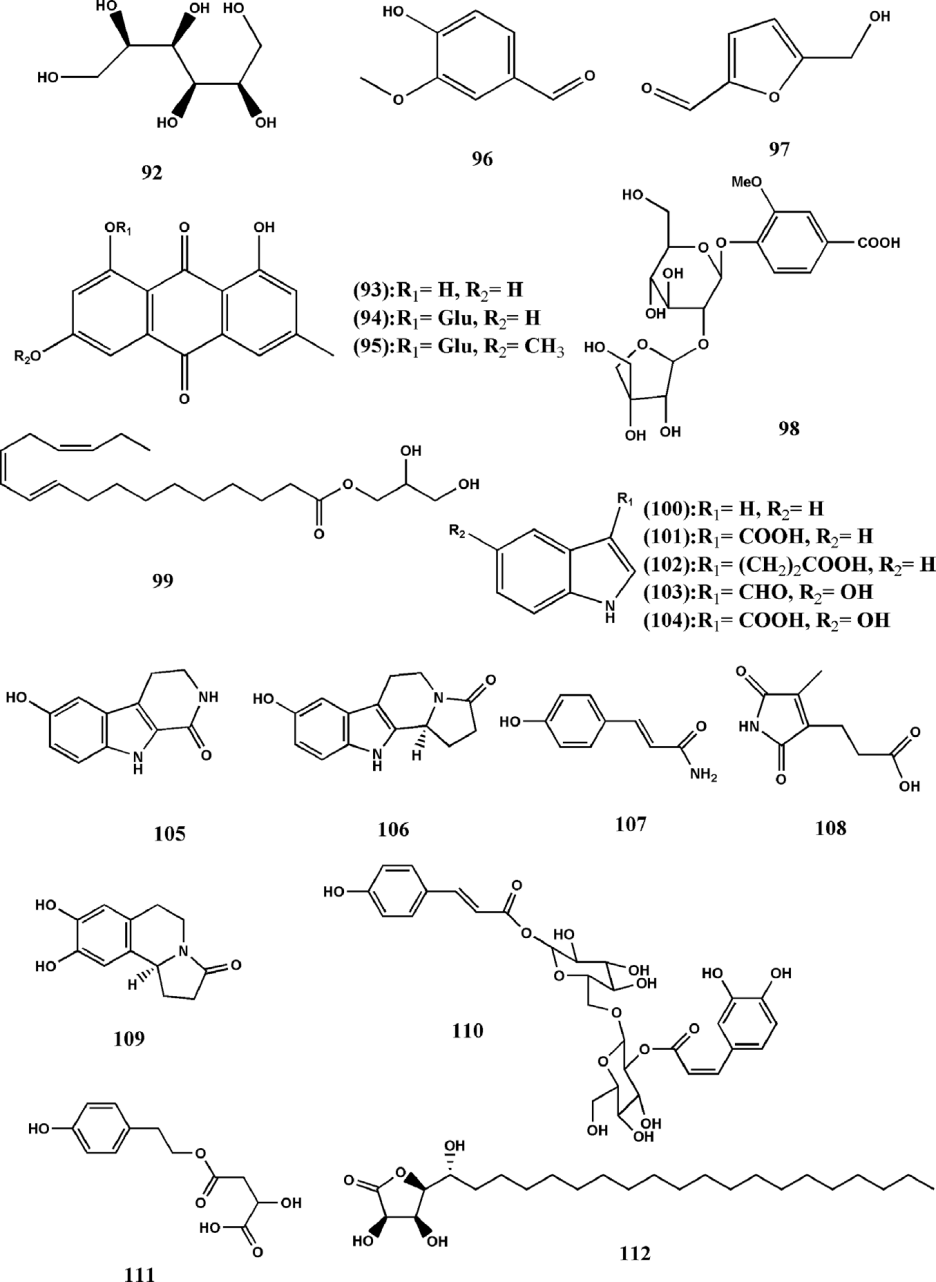
Structures of the anthraquinones, alkaloids, and other components from *Tetrastigma hemsleyanum* Diels & Gilg. m *Tetrastigma hemsleyanum* Diels & Gilg.

According to the existing reports, it can be seen that there are other chemical components in TDG, among which polysaccharide is regarded as necessary active one because of its various biological activities. Rao et al. (2016) determine the contents of fucose, rhamnose, arabinose, mannose, glucose, and galactose in the polysaccharide by ion chromatography. And Shao et al. (2011) use response surface methodology to optimize the extraction process of polysaccharides from three different factors of TDG from. These studies have laid the foundation for the subsequent research on polysaccharides from TDG. In addition to polysaccharides, Fu et al. (2012) conduct inductively coupled plasma optical emission spectrometry (ICP-OES) research and find that there are many trace elements in TDG, and the higher contents are Mg, Fe, Mn, Zn and Ba, and so on, in turn. In the follow-up studies, they discover selenium (Fu J. et al., 2017). Cai et al. (2013) result in the existence of amino acids and cardiac glycosides through the characteristic identification reaction, but there are few related researches. Besides, there are other substances in TDG, for example, mannitol (92), emodin (93), emodin-8-O-β-D-glucopyranoside (94), physcione-8-O-β-D-glucopyranoside (95), 4-hydroxy-3-methoxybenzaldehyde (96), 5-hydroxymethyl furfural (97) (Liu, 2000; Zeng, 2013; Ding et al., 2015b). With the continuous development of science and technology, Liu et al. (2015) extracts a new phenolic glycoside from TDG tuber, designs it as hemsleyanumoide (98), and determines its specific structure through various methods. Jin et al. (2018) first discover a new polyunsaturated fatty acid glyceride in TDG, namely (9R)-Hydroxy-(10 E, 12Z, 15Z)-octadecatrienoic-2, 3-dihydroxypropyl ester (99). Separation of the EtOAc-soluble fraction of the methanolic extract of the aerial parts of TDG result in the isolation of ten alkaloids. Then, Wang et al. (2018a) also discover that with the spectral data in the current literature, and the alkaloids are identified as indole (100), indole-3-carboxylic acid (101), indole-3-propanoic acid (102), 5-hydroxy-indole-3-carboxaldehyde (103), 5-hydroxy-indole-3-carboxylic acid (104), 6-hydroxy-3,4-dihydro-1-oxo-β-carboline (105), hippophamide (106), 4-hydroxycinnamide (107), pyrrole-3-propanoic acid (108) and S-(-)-trolline (109). In addition, three new compounds are isolated from the ethyl acetate fraction of the 90% EtOH extract of TDG aerial parts, which are 1-O-trans-p-hydroxycinnamoyl-2’-O-trans-caffeoyl gentiobiose (110), 2S-hydroxy-4-(4-hydroxy-phenethoxy)-4-oxobutanoic acid (111), and (3R, 4S, 5R) -3, 4-dihydroxy-5-((R) -1-hydroxyeicosyl) dihydrofuran-2 (3H)-one (112). Their structures are elucidated by UV, IR, one- and two-dimensional nuclear magnetic resonance, high-resolution mass spectrometry data, and other methods. The preliminary researches have shown that compounds (110, 111) have a weak inhibitory activity against soluble epoxide hydrolase, while compound (112) shows moderate cytotoxicity against the HCT116 cell line (Wang et al., 2018b) (structure can be seen in **Figure 6** and the names of the compounds are listed in **Table 5**).

**Table 5:** Anthraquinones, alkaloids, and other components from *Tetrastigma hemsleyanum* Diels & Gilg.

No.	Name	Formula	Molecular Weight	[M-H]^-^ or [M+H]^+^ (m/z)	MS/MS fragments (m/z)	References
**92**	Mannitol	C_6_H_14_O_6_	–	183	183 (1), 165 (1), 133 (10), 103 (50), 73 (100)	(Liu, 2000)
**93**	Emodin	C_15_H_10_O_5_	–	269 (−)	–	(Zeng, 2013)
**94**	Emodin-8-O-β-D-glucopyranoside	C_21_H_20_O_10_	–	431 (−)	–	(Zeng, 2013)
**95**	Physcione-8-O-β-D-glucopyranoside	C_22_H_22_O_10_	–	–	–	(Zeng, 2013)
**96**	4-hydroxy-3-methoxybenzaldehyde	C_8_H_8_O_3_	–	168 (+)	–	(Ding et al., 2015b)
**97**	5-hydroxymethyl furfural	C_6_H_6_O_3_	–	126 (+)	–	(Ding et al., 2015b)
**98**	Apigenin-6-C-α-L-arabinopyranosyl-(1-4)-α-L-rhamnopyranoside	C_19_H_26_O_13_	–	461.1303 (−)	–	(Liu et al., 2015)
**99**	(9R)-Hydroxy-(10 E,12Z,15Z)-octadecatrienoic-2,3 -dihydroxypropyl ester	C_21_H_36_O_4_	–	369.2637 (+)	–	(Jin et al., 2018)
**100**	Indole	C_8_H_7_N	–	–	–	(Wang et al., 2018a)
**101**	Indole-3-carboxylic acid	C_9_H_7_NO_2_	–	–	–	(Wang et al., 2018a)
**102**	Indole-3-propanoic acid	C_11_H_11_NO_2_	–	–	–	(Wang et al., 2018a)
**103**	5-hydroxy-indole-3-carboxaldehyde	C_9_H_7_NO_2_	–	162.0553 (+)	–	(Wang et al., 2018a)
**104**	5-hydroxy-indole-3-carboxylic acid	C_9_H_7_NO_3_	–	–	–	(Wang et al., 2018a)
**105**	6-hydroxy-3,4-dihydro-1-oxo-β-carboline	C_11_H_10_N_2_O_2_	–	203 (+)	–	(Wang et al., 2018a)
**106**	Hippophamide	C_14_H_14_N_2_O_2_	–	265.0950 (+)	–	(Wang et al., 2018a)
**107**	4-Hydroxycinnamide	C_9_H_9_NO_2_	–	–	–	(Wang et al., 2018a)
**108**	1H-pyrrole -3-propanoic acid	C_8_H_9_NO_4_	–	–	–	(Wang et al., 2018a)
**109**	S- (-)-trolline	C_12_H_13_NO_3_	–	219 (+)	–	(Wang et al., 2018a)
**110**	1-O-trans-p-hydroxycinnamoyl-2′-O-trans-caffeoyl gentiobiose	C_30_H_34_O_16_	–	649.2132 (−)	–	(Wang et al., 2018b)
**111**	2S-hydroxy-4- (4-hydroxyphenethoxy) -4-oxobutanoic acid	C_12_H_14_O_6_	–	253.0938 (−)	–	(Wang et al., 2018b)
**112**	(3R, 4S, 5R) -3,4-dihydroxy-5-((R) -1-hydroxyeicosyl) dihydrofuran- 2 (3H) -one	C_24_H_46_O_5_	–	–	–	(Wang et al., 2018b)

“-”: Some relevant information in the literature is incomplete.

(−): [M-H] ^-^ (m/z).

(+): [M+H] ^+^ (m/z).

## Pharmacological Activities

### Anti-Tumor Activity

Cancer is one of the most fatal diseases in the world. With complex causes, it involves multiple pathways and is easy to produce drug resistance after administration. In clinical treatment, western medicine remains a top priority in treatment, but some serious side effects will also cause some physical and mental injury to patients. Therefore, many scholars have focused on natural drugs and screened active ingredients with anti-tumor properties, such as Isoxazolines contribute significantly in anti-cancer activity against cancer, and most of the Isoxazoline are found in natural products, these compounds have broad-spectrum therapeutic implications because of structural specificity (Kaur et al., 2014). As a precious Chinese herbal medicine, TDG has been found to show obvious anti-tumor effects both *in vivo* and *in vitro*. Therefore, we summarize the research on the anti-tumor effect of TDG in recent years.

Nowadays, many studies have shown the anti-tumor effect of TDG. Earlier tests show that TDG extract has a significant inhibitory effect on A549 cells *in vitro*, which is positively related to the concentration and duration of the drug, and might be related to the expression of BcL-2 gene (Cheng and Lu, 2007). Moreover, the extract of TDG has a certain inhibitory effect on the tumor in S180 tumor-bearing mice. As a result, only after the intervention of the high dose group, it finds that T lymphocyte subpopulations and antioxidant indexes are significantly changed and these findings suggest that the anti-tumor mechanism of TDG may be related to the improvement of immune function and activation of the antioxidant pathway (Xu et al., 2009). A study shows that ethanol extract of TDG in a certain concentration range (1 to 625 μg/ml) could inhibit the proliferation of A375 cells in a dose-dependent relationship, and affect its tyrosinase activity and Synthesis of melanin (Lv et al., 2011). In addition, Li and Peng (2014) discover that TDG extract can induce apoptosis of human cervical cancer Hela cells in a concentration- and time-dependent manner. As shown by continuous researches, different extraction parts have certain influence on the anti-tumor activity of the extract but with different effect. Ding et al. (2005) compare various extracts from TDG through *in vitro* experiments of liver cancer cells HepG2 and primary rat hepatocytes, namely ethyl acetate part, n-butanol part, petroleum ether part, and water soluble part. The effect of the extracts from different parts on normal cells is smaller than that of tumor cells, and the ethyl acetate-soluble portion takes a strong inhibitory effect on the activity of human liver cancer cells HepG2. Different extraction sites may have a certain inhibitory effect on tumor cells, so the study has found that the anti-tumor active positions in TDG are chloroform and petroleum in view of tumor suppression rate (Ding and Ji, 2011). Moreover, related studies also exhibit that ethyl acetate TDG extract has obvious anti-tumor effects, with its molecular mechanism possibly involved in immune-related factors and pro-apoptotic proteins (Wang et al., 2014; Zhong et al., 2014). Above all, preliminary studies have shown the effect of TDG on anti-tumor. Xiong et al. (2015) develop gas chromatography-mass spectrometry (GC-MS) method to analyze the chemical constituents of petroleum ether fraction of TDG, and then find that petroleum ether fraction of TDG inhibits the growth and induces apoptosis of HeLa cells in dose-dependent and time-dependent manner. The mechanism may be triggered by intrinsic apoptotic pathway indicated by the loss of mitochondrial membrane potential and the activation of caspase-9 and caspase-3, and extrinsic apoptotic pathway indicated by the activation of caspase-8. In another study, ethylene extract of TDG has certain cytotoxicity to liver cancer cells, which may be caused by the induction of S phase arrest and apoptosis of HepG2 cells, then, subsequent shows that ethylene extract of TDG induces apoptosis of HepG2 cells by triggering the mitochondrial caspase-dependent intrinsic pathway rather than the death receptor (Peng et al., 2015; Peng et al., 2016c). On this basis, the same extract about the previous sentence from TDG inhibits the proliferation and colony formation of HepG2 and SMMC-7721 cells, while regulating apoptosis *via* the Caspase family and Bcl-2 gene family signaling pathways (Chen et al., 2018). These findings all demonstrate that ethylene extract of TDG has a better inhibitory effect on tumor cells, but the mechanisms involved are not entirely consistent. However, none of these authors have proved the specific medicinal ingredients and there is lack of positive control. The anti-proliferative activities of four extracts (CHCl_3_, EtOAc, n-BuOH, and H_2_O) are compared through *in vitro* experiments. The results show that CHCl_3_ extract has the highest activity, with 10 compounds which are further isolated. Preliminary studies have displayed that that resveratrol and kaempferol exhibit obvious inhibitory effects on MDA-MB-435S cells (Lin et al., 2016), which need to be further studied to provide strong evidence.

TDG has complex chemical components, mainly flavonoids and phenolic acids. Current researches on the anti-tumor effect of TDG have focused on aspects of the respiratory system, digestive system, reproductive system, and so on. In the *in vitro* experiments with different lung cancer cells (A549 cells and A431 cells), the total flavonoids of TDG can inhibit the proliferation and apoptosis of tumor cells, and its mechanism of action involves the Bcl-2 family proteins, Caspase family proteins, MAPK pathway, and the ubiquitin-proteasome pathway. Besides, the total flavonoids of TDG can markedly inhibit the metastasis and invasion of tumor cells, with migration and invasion being associated with the activation of MMPs and TIMPs proteins (Zhong et al., 2013; Zhong et al., 2016; Zhong et al., 2017; Zhong et al., 2019). The above studies indicate that the total flavonoids of TDG has a very good therapeutic effect on lung cancer at the cellular level. The miRNA profile of A549 cells treated with total flavones of TDG is established, and the change of miRNA expression profile is determined by using miRNA-seq analysis. As a result, 162 miRNAs have been identified to display expression changes >1.2-fold in RTHF-treated A549 cells. On the basis of the above research, Liu P. et al. (2019) also find that miR-4792 may be involved in the inhibition of A549 cell proliferation and metastasis mediated by total flavones of TDG. Meanwhile, total flavones of TDG is to down-regulate the overexpression of miRNA in cancer, and also up-regulate the expression of tumor suppressor miRNAs. In addition, according to experimental results, total flavonoids of TDG can induce cell apoptosis by modulating some apoptotic-related proteins. Similarly, Feng et al. (2006) find in SGC-7901 gastric cancer cells that total flavones of TDG inhibit tumor growth, invasion, and metastasis by down-regulating MMP-2 expression. A study shows that total flavonoids of TDG can inhibit the proliferation of HepG2 cells treated by PGE2, and also offset activator of EP2 receptor-induced proliferation of HepG2 cells. Meanwhile, the mRNA expression and protein expression associated with the COX-2-Wnt/β-catenin signaling pathway are changed by the methods of PCR and Western Blot. The total flavonoids of TDG can promote the apoptosis of HepG2 cells through *in vitro* and *in vivo* experiments and have a positive correlation with the drug concentration (Li et al., 2017). A similar study exhibits that the total flavonoids of TDG may inhibit Wnt/β-catenin signaling pathway activation, thereby inhibiting multiple malignant biological behaviors of colorectal cancer (Wu et al., 2018). Furthermore, the total flavonoids of TDG can also dose dependently induce cell cycle arrest at G0/G1 phase and inhibit epithelial-mesenchymal transition process (Xia et al., 2018). Qiu et al. (2019) find that the total flavones of TDG can inhibit the proliferation of different breast cancer cells, which may be induced by apoptosis by up-regulating the expression of Caspase-3. And they can inhibit cell proliferation in MDA-MB-468 and MCF-7 cells by inhibiting the expression of p-p42/44 and blocking the MAPK signaling pathway. Previous studies have suggested that aberrant activation of hepatocyte growth factor/scatter factor (HGF/SF) and its receptor, Met, is involved in the development and progression of many human cancers. A flavonoid from the root of TDG, Isoquercitrin (quercetin3-O-b-D-glucopyranoside), and find that inhibits (HGF/SF met) signaling by decreasing the amount of tyrosine phosphorylation in human cancers (Xia et al., 2018), but the specific mode of action is not clear. In another study, Isoquercetin is obtained by selective enzymatic derhamnosylation of rutin using recombinant a-L-rhamnosidase from *Aspergillus terreus*, and the mechanism of its effect on human ovarian cancer cells is analyzed. After treatment with Isoquercetin, it can inhibit the production of SOD in the cell, and the concentration cannot be too high, otherwise it would have an adverse effect. These findings prove that the protective effect of Isoquercetin on ovarian cancer cells may be mediated through the antioxidant pathway (Michalcova et al., 2019). Some studies have shown that the anti-tumor activity of TDG may be associated with the suppression of immune-related factors. Further research shows that TDG flavonoids can dramatically reduce the serum levels of TGF-β, PGE2, and cyclooxygenase-2 in tumor bearing mice, thus hindering the development of regulatory T cells (Feng et al., 2014a; Feng et al., 2014b). There are many phenolic compounds in the roots of TDG. Sun et al. (2017a) purify solid 80% methanol crude extracts to obtain total phenol extracts and flavonoid extracts by solid-state extraction, and identifies and determines 24 individual phenolics in the extract *via* chemical analysis, as well as investigating their anti-tumor effect in H22 tumor-bearing mice. The test results can show that the content of the semi-purified extract is higher than that of the crude extract. In addition, the extract increases the production of cytokines (TNF-α and IL-2) in the serum, the CD4/CD8^+^ ratio and the level of NK cells, while regulating the expression of related proteases, as well as inhibiting the expression of vascular endothelial growth factor (VEGF). These studies indicate that solid-state extraction technology can better retain effective ingredients, and the anti-tumor mechanism of TDG extract may be related to induction of apoptosis, inhibition of angiogenesis, immune function, and antioxidant activity. Most of the above studies focus on the underground part of TDG, and the research on the aerial part of TDG has also made some progress. Sun C. et al., (2018) and Sun Y. et al. (2018) study the phenolic acid components contained in TDG leaves extract with LC-QTOF-MS/LC-QqQ-MS techniques, and discovers that the main phenolic components are apigenin and luteolin glycosides, while exploring TDG extract anti-hepatic cancer mechanism. Experiments indicate that TDG leaves extract inhibits growth and induces apoptosis of liver cancer cells through the internal mitochondrial signaling pathway and the external death receptor signaling pathway Sun (2018). The above researches strongly prove that TDG has its unique advantages in anti-tumor function, so it has a broader prospect to develop and utilize the anti-tumor medicinal value TDG. However, the structure-activity relationship between active ingredients and anti-tumor activity, as well as the metabolism of active ingredients *in vivo* has not been understood so far.

### Anti-Oxidative Activities

Oxidative stress is one of the causes of many pathological diseases, such as cancer, chronic diseases, and cardiovascular diseases. In a study, the total phenolic components in the leaves of tropical medicinal plant TDG from Sabah have different degrees of antioxidant capacity due to the different extraction solvents, in the order of methanol extract > ethyl acetate extract > chloroform > butanol > hexane extract (Hossain et al., 2011). Sun et al. (2013, 2015) primitively study the phenolic compounds—especially flavonoids—which are present in TDG leaves or tubers, and proves their good antioxidant activity by chemical based antioxidant tests (DPPH, ABTS, FRAP assays). The test results show that five kinds of flavonoids (catechin, kaempferol-3-rutinoside, rutin, isoquercitrin, and astragalin) may be the main antioxidants. Moreover, oral administration with TDG in rats suggests that phenolic compounds and their metabolites in the leaf of TDG extract are also bioavailable, and its mechanism of action involves the favorable changes in several antioxidant biomarkers and lipid peroxidation product (Sun et al., 2017a; Sun et al., 2017b). Ye and Liu (2015) optimize the extraction process of total flavonoids from TDG, and come to a conclusion that flavonoids have remarkable antioxidant and free radical scanning activity. However, the molecular mechanism of anti-oxidation of TDG has not been mentioned in the above studies, so it is necessary to carry out experimental studies in the future. Wang et al. (2016) establish in a rat model with chronic obstructive pulmonary disease (COPD) to explore the protective effect of TDG and its mechanism. The levels of IL-8 and CRP in serum and bronchoalveolar lavage fluid (BALF) are detected by ELISA, while reducing local MDA levels and increasing superoxide dismutase (SOD) levels in both pulmonary tissue homogenate and BALF, and detected mRNA and protein expression of nuclear factor-carotenoid 2 related factor 2 (Nrf2), are all up-regulated. These effects suggest that the protective effect of TDG may be related to its anti-inflammatory and anti-oxidative capacity through Nrf2 activation, but further research is required. Chu et al. (2019b) extract purified polysaccharide from TDG (TVP) with 64.89 kDA, and identify its structural composition by various analytical methods. Then the oxidative stress models induced by EC are constructed in Caco-2 cells and *C. elegans*, results show that TVP could attenuate EC (a carcinogen) -induced cytotoxicity. In this study, three nematode models have also been compared, and daf-16 (−) mutant is assessed to be related to the antioxidant effect of TVP. More data are demanded to support this conjecture. Future research should attempt to find a suitable and available animal model to study the antioxidant activity of TDG extract *in vivo*, which will be more conducive to comprehensively explain the relevant mechanism of its antioxidant activity.

### Anti-Inflammatory, Antipyretic, and Analgesic Effects

Huang et al. (2005) adopt different animal models, and the test results show that TDG extract has strong anti-inflammatory, analgesic, and antipyretic effects, providing scientific evidence for its clinical application. Liu et al. (2016) confirm that the flavonoids from TDG (10–160 μg/ml) can reduce the excretion of pro-inflammatory factors and increase the production of anti-inflammatory cytokines in LPS induced cell inflammation model, and find that the flavonoids from TDG can reverse the up-regulation of TLR4, MD-2, MyD88, and TLR4/MD-2 complex expression, so as to affect photosynthesis and activity of TLR4/MD-2 mediated NF JB and JNK pathway. Similar researches have shown that the flavonoids from TDG ameliorates inflammation through JNK, p38, and Nrf2 pathways, inhibits oxidative stress reactions caused by morphological (both cell and nucleus) changes and inflammation. In addition, the flavonoids from TDG has good biological properties in anti-inflammatory, cytoprotective, and anti-aging active (Li Y. et al., 2019). Ji et al. (2019) establish a ConA-induced mouse hepatitis model to investigate the anti-inflammatory effects of total flavonoids in TDG, taking bifendate as a positive control, which turns out that pretreatment with total flavonoids from TDG can reduce the serum levels of ALT and AST, pro-infammatory (IL-17 and IL-6) in serum and the proportions of Th17 cells in spleen. In the meantime, pretreatment with total flavonoids from TDG could increase the percentage of Treg cells in spleen and the levels of transforming growth factor (TGF)-β1, IL-10 in serum. Furthermore, Foxp3 and RORγt are liver-specific transcription factors, respectively, an essential transcription factor in the development of Treg cells or Th17 cells. The experimental results show that the expression of Foxp3 increases while that of RORγt decreases. The effect is mediated by regulating Treg/Th17 immune homeostasis. However, some of these results did not design *in vivo* to evaluate their efficacy. However, some of these results were not designed *in vivo* to evaluate their efficacy. In addition to total flavonoids from TDG, the polysaccharide from the root have been confirmed as the main active components of the antipyretic and anti-inflammatory of TDG. The results show that the polysaccharide has a strong antipyretic and anti-inflammatory effect through the experiments of different animal models, but the mechanism of its action is not clear (Ding et al., 2017). Chu et al. (2019a) isolate and extract a kind of polysaccharide (TTP-1) with an average molecular weight of 478.33 kDa from the root of TDG tuber, whose results indicate that TTP-1 could attenuate inflammation *via* COX2, iNOS, MAPKs pathways and ameliorate oxidative damage *in vitro* through Nrf2-Keap1, Sirt1-FoxO1 pathways in RAW264.7 cells. On the other hand, *in vivo*, TTP-1 improves the growth and development of Caenorhabditis elegans triggered by LPS-induced inflammation, exercise capacity, and clears ROS and O^2-^. In a word TTP-1 exhibits excellent anti-inflammatory and anti-oxidant ability both *in vitro* and *in vivo*. However, there is no positive control in this study. Liao et al. (2017) discover that the aerial part of TDG has antipyretic, analgesic, and anti-inflammatory effects, and the efficacy increases with the increase of drug dosage, but the molecular mechanism by which this activity happens should be further studied. Wang et al. (2018a) isolate alkaloids from the aerial part of TDG for the first time, three of which have good anti-inflammatory activity. Besides, the results demonstrate that the crude extract of aerial part of TDG attenuate the phosphorylation of three major MAPKs (JNK, ERK, and p38), whereas S-(-)-trolline inhibits only the activity of ERK-MAPK, thereby inhibiting induction of inflammatory cytokines or mediators such as interleukin-1β (IL-1β) and inducible nitric oxide synthase (iNOS).

### Antiviral Effect

According to the existing literature, the extract of TDG also has important pharmacological activity in terms of anti-virus. The four extracts of TDG exert inhibitory effect on hepatitis B virus (HBV), especially the ethyl acetate part, so this extract can significantly reduce the level of DNA replication of HBV in serum (Yang and Wu, 2009). These extracts have been found to possess certain inhibitory effects on different HIV-1 virus strains *in vitro*, but the mechanism of action is unclear (Dong and Li, 2016). Pr8-ns1-gluc method is established for quantitative analysis of 10 compounds in 18 batches of gastrodia eluga, and HPLC-MS method and pr8-ns1-gluc method are used to detect the biological activity of the above samples against influenza virus. As a result, it is shown that the content of chemical components of TDG varies with the change of producing area, with the antiviral activity of different batches changing accordingly, as well as the antiviral activity of different batches altering as well. Among those chemical compositions, eight flavonoids are positively correlated with anti-H1N1 virus activity (Ding et al., 2019). Studies on the antiviral effects of TDG are confined only to *in vitro* experiments, and lack of animal models to evaluate the pharmacodynamic action of TDG *in vivo*.

### Hepatoprotective Effect

Yang (2008) utilize bacillus Calmette-Guérin (BCG) and lipopolysaccharide (LPS)-induced animal models of immune liver injury in mice to study the protective effect of TDG on immune liver injury, and detected the changes of alanine aminotransferase (ALT), aspartate aminotransferase (AST), lactate dehydrogenase (LDH) in serum, as well as the lipid peroxidation products malondialdehyde (MDA) and superoxide dismutase (SOD) in liver tissues. The transformations of the above indicators prove that the liver injury is alleviated to some extent. Moreover, in some studies, both total amino acids and polysaccharide components in TDG exert certain protective effects on acute liver injury induced by carbon tetrachloride (CCl_4_) in mice (Huang and Mao, 2007; Ma et al., 2012). However, these experiments are unable to elucidate the molecular mechanism of hepatoprotective effect of TDG. The treatments of TDG given to rats with chronic liver injury induced by CCl_4_ reduce the levels of ALT, AST, and total bilirubin (T-Bili) in blood serum. Apart from that, the administration of TDG also decreases the expression levels of total protein (TP), albumin (ALB) as well as numerical value of A/G. These finding suggest that TDG has a good protective effect on the liver. Laminin (LN) and hyaluronic acid (HA) are considered as the indicators of liver fibrosis, with their changes reflecting the degree of liver lesions. TDG could notably inhibit the abnormal increase of HA and LN, revealing that TDG possesses a remarkable anti-fibrosis effect (Zhang and Ni, 2008). Previous researches have indicated that the superfine particles of TDG have hepatoprotective activity. The superfine particles of TDG obtained through a novel superfine particle processing technique, and the results show that the superfine particles of TDG hinder the up-regulation of ALT and AST levels, reduce MDA levels, and decrease the expression of Bax and caspase-3 proteins. Moreover, the superfine particles of TDG enhances SOD activity and apparently improves histopathological lesions. These investigations suggest that the hepatoprotective effects of SPRT are likely connected to the free radical scavenging effect (Cao et al., 2014). A preliminary exploration is carried out in hepato-protective effects of TDG, which is commonly used by treating of hepatitis and liver fibrosis in the clinic, but little is known about the therapeutic material basis and related mechanism of action.

### Immunomodulatory Effects

Xu et al. (2008) make research on the effect of ethyl triacetate fraction (EAF) isolated from TDG on the immune function of ICR mice. The results prove that EAF isolated from TDG is able to increase the mouse spleen lymphocyte transformation induced by concanavalin A (ConA), the delayed type hypersensitivity (DTH) in dose-dependent manner, as well as significantly enhancing the ability of macrophages to phagocytose India ink. These findings suggest that the mechanism of this extract might be related to elevate levels of serum interferon-gamma (IFN-γ) and serum tumor necrosis factor-alpha (TNF-α), and strengthen the effect of mononuclear–macrophage phagocytosis. In addition, Zhu et al. (2020) make use of multiple analytical methods to characterize the chemical element of a purified polysaccharide extracted from the aerial part of TDG. The experimental results show that the aerial part of TDG is composed of galacturonic acid (GalA), glucose (Glc), mannose (Man), arabinose (Ara), galactose (Gal), and rhamnose (Rha), and it has an average molecular weight of 66.2 kDa. By establishing the yeast-induced hyperthermia mice models, the authors find that the aerial part of TDG may be related to the concentration of PGE2, cAMP, and IL-6 in serum with notable antipyretic effect. In addition, the spleen index, thymus index, and the number of spleen cells enhance with the increase of the aerial part of TDG concentration in H22 tumor-bearing mice. After the addition of TLR4 inhibitor TAK242 in LPS-induced cell model, the cytokine level decrease, indicates that the aerial part of TDG may compete with TLR4 in interacting with TLR4 to regulate the production of cytokines, thereby regulating immune function and achieving anti-tumor activity. In one study, different TDG tuber extracts could promote the up-regulation of pore-forming protein (PFP), Granzyme B (GrB), CD107a, and IFN-γ expression on NK cell surface, and the best effect is obtained by boiling boiled sugar (Wang et al., 2018c). In conclusion, TDG contributes significantly to enhancing the immune function, and prevents inflammation, cancer, and other diseases. However, little research has been done on the immune activity of TDG.

### Other Activities

In addition to the above pharmacological activities, a recent study shows that a water-soluble polysaccharide with a molecular weight of 93307 Da, named THP, is extracted from TDG. THP has exhibited strong hypoglycemic activity in the alloxan induced mouse model, with its treatment being discovered to promote the activity of antioxidant enzymes (GSH-Px, SOD, and CAT) and reduce the content of malondialdehyde (MDA), while THP could restore the structure of pancreas and therefore affect insulin release, as it may be a natural candidate for the treatment of diabetes (Ru et al., 2018). The research team obtained novel of polysaccharide (THDP-3) purified from the stems and leaves of TDG *via* anion exchange chromatography with a molecular weight of 77.98 kDa. The structural analysis of the compound show that THDP-3 is composed of rhamnose, arabinose, mannose, glucose, and galactose, with a molar ratio of 1.0:1.3:2.5:2.3:3.1. The same is that both THDP-3 and THP have obvious hypoglycemic effects. Experimental results indicate that THDP-3 may be able to regulate the expression of glucokinase, ampactivated protein kinase, glucose-6-phosphatase, and phosphoenolpyruvate carboxykinase, of which involving in glycogen metabolism pathway. These new findings suggest that THDP-3 can promote glycogen synthesis and inhibit gluconeogenesis (Ru et al., 2019b). Moreover, a new polysaccharide TDGP-3 from TDG with a molecular weight of 3.31 × 10^5^ Da by adopting the enzyme-ultrasonic assisted extraction (EUAE) method. The treatment of TDGP-3 could increase the activity of antioxidant enzymes in the liver at a dose of 300 mg/kg, and lowers blood lipid levels (TC, TG, HDL-C, and LDL-C) and MDA in the liver (Ru et al., 2019a). These effects suggest that TDGP-3 can effectively reduce blood lipid and antioxidant activity. The molecular target mechanisms combined with the hypoglycemic and lipid-lowering activities of the polysaccharides of TDG are not delved into by researchers. Furthermore, Ding et al. (2018) develop a preparation method to obtain safe and non-toxic oligosaccharides in TDG with an extraction rate of 1.586%, and anti-tumor pharmacological experiments are found *in vivo* to improve intestinal flora and protect the gastrointestinal tract and so on. The investigation has suggested that TDG polysaccharide could inhibit the growth of E. coli and possessed prominent antibacterial activity. Chen et al. (2019) employ a method of metabolomics combined with HPLC/MS and find that TDG’s polysaccharide interferes with the conversion of F6P to FBP and inhibits the E. coli’s proliferation. Chu et al. (2020) explore the protective mechanism of TDG leaves extract against ACR induced toxicities in HepG2 cells and *Caenorhabditis elegans* (*C. elegans*). They find that TDG leaves attenuated ACR induces HepG2 cytotoxicity and prevents HepG2 cells from oxidative stress induced by ACR *via* regulating Akt/mTOR/FoxO1/MAPK signaling pathway. Five main compounds are identified from TDG leaves, among which 5-caffeoylquinic acid (5-CA) is proved to be part and parcel in improving the toxicity of ACR in TDG leaves. Furthermore, TDG leaves improves survival rate, viability, growth and development ability, and anti-oxidative stress ability of N2 *C. elegans*. According to qRT-PCR analysis, TDG leaves could upregulate the expression of antioxidant related genes by influencing transcription factor DAF-16. It is discovered that TDG extract can inhibit the proliferation of leukemia K562 cells. By promoting the high expression of the tumor suppressor gene P53 and the low expression of the oncogene C-myc, the dual effects of inhibiting cell proliferation and promoting cell apoptosis are demonstrated (Xu et al., 2010). Wang C. et al. (2019) isolate 39 kinds of compounds from the extract of TDG, among which kaempferol and apigenin exert remarkable inhibitory activities against soluble epoxide hydrolase (sEH) and inducible nitric oxide synthase (iNOS). Based on the structure analysis of the compound, the number of OH groups in the benzene ring of the tested compounds may contribute to the increase in sEH inhibitory activity, and the sugar unit in the flavone glycoside is unfavorable for efficient inhibition of NO production. Both sEH and iNOS are related to the function of blood vessels, meanwhile, kaempferol and apigenin may act as dual inhibitors of sEH and iNOS. It can be seen from these studies that TDG is conducive to treating cardiovascular diseases, but the mechanism of this effect needs to be further studied to provide evidence for.

### Short for the Pharmacological Mechanism of TDG

According to existing reports, this event that TDG has a good anti-tumor activity was confirmed. The possible anti-tumor mechanisms involved in this action can be divided into the following five sectors. First, TDG can induce apoptosis of tumor cells, thereby achieving anti-tumor effects. And this process, mainly involved in the apoptosis pathway includes the death receptor pathway and the mitochondrial pathway, which ultimately leads to the occurrence of apoptosis. For instance: In the extrinsic death receptor pathway, TDG may be connected with the related protein expression (Fax/FasL, FADD) on the surface of liver cancer cell membranes, after binding to this protein receptor, it forms a complex, which affects the increased expression of some proteins in the caspase family and eventually leads to apoptosis. In the mitochondrial signaling pathway, TDG can promote the secretion of cytochrome C, further lead to the cleavage of PARP, and which affects the function of mitochondria. At the same time, TDG also can affect the Bcl-2 gene family Bcl-2 family proteins and MAPK pathway. These changes ultimately result in DNA damage and promoted cell apoptosis (Zhong et al., 2013; Sun Y. et al., 2018). Second, TDG can improve immune function by increasing the production of immune-related factors and affecting the function of T lymphocytes. Third, TDG will reduces vascular endothelial growth factor (VEGF) level and inhibits tumor vascular growth. Fourth, TDG can eliminate free radicals produced in tissues or cells due to external factors and prevent chronic diseases and cancer caused by oxidative damage. Five, TDG can inhibit the proliferation, metastasis, and invasion of tumor cells, which may be involved in the ubiquitin-proteasome system (UPS). Studies on the antioxidant activity of TDG have mainly found that TDG may change local MDA levels, superoxide dismutase (SOD) levels, and the expression levels of related factor proteins. However, few studies have been carried out on anti-inflammatory, antipyretic and analgesic effects, antiviral effect, hepatoprotective effect, immunomodulatory effects of TDG. TDG has been discovered about the complex biological activities they displayed, some mechanisms of action of TDG remains unclear and demands further inspection.

## Quality Control

### Cultivation and Processing Technology

Because of the increasing shortage of wildlife sources, cultivated method starts to be applied on TDG to meet the market demand in consideration of the low survival rate of TDG, so it is of significant to know the growth conditions of TDG, select good varieties, and develop appropriate cultivation techniques. A study discovers that TDG possesses low light acclimation capacity, with the optimal light irradiance condition for TDG cultivation being approximately 67% of the shade. Simultaneously, TDG’s growth and development are limited by ambient temperature (Dai et al., 2009). From the transcriptome analysis, Peng et al. (2019) conclude that a total of 114 single genes are assigned to the flavonoid biosynthetic pathway, along with the expression of genes related to flavonol biosynthesis and flavonol content increased in TDG under cold stress. These findings provide valuable information regarding to the transcriptome changes in response to cold stress, and may be useful for creating the novel germplasms with high cold-tolerance *via* molecular breeding. The complete genomic sequence of TDG has been reported to enrich TDG plant information (Li et al., 2016). Song et al. (2017) isolate 31 endophytic fungi belonging to 10 genera and add fermented broth of endophyte strains (named TH09, TH12, TH14, TH15, TH17, and TH26 respectively) to MS medium to culture axenic seedlings. After 30 days of culture, it is found that endophytic fungi can promote the growth of host plants by measuring net growth, expansion gene expression and flavonoid content, as well as upregulating expansion gene expression and increasing the medicinal ingredient flavonoid synthesis. These findings indicate that new methods for cultivation can be developed from the microbiological aspect to cultivating high-quality varieties. By comparing water extract and alcohol extract, Liu Y. et al. (2019) evaluate the differences in the various biological activities of the two extracts through different cell models, and eventually get the result that the ethanol extract has good antioxidant activity and decreases with increasing temperature. Whether it is water extract or alcohol extract, research has shown that the temperature should not be too high, otherwise it would destroy its effective ingredients.

Processing is a common step in TCM treatment of medicinal materials, which can improve the effectiveness of clinical medication. However, different processing methods may bring different effects, such as reducing toxicity, improving efficacy, changing drug performance, reducing irritation, and other results. Therefore, TDG should be processed in order to improve medicinal effect. Its traditional processing methods mainly include low temperature drying into powder, hot air drying after slicing, and decoction as soup, especially the latter two methods which are related to temperature. In the process of operation, it is easy to find the loss of effective ingredients, and at the same time, there are differences in different parts of the medicinal materials. Therefore, the related research aims at the above-mentioned problems and takes some measures to maximize the retention of active ingredients. For example: developing a highly active oral TDG micropowder, using different processing methods for the active ingredients contained in different parts, making medicine power into granules (Cheng et al., 2015; Lang, 2019). At present, the research on the cultivation and processing technology of TDG is still very weak, so it is necessary to further enlarge the investment in research and improve the cultivation and processing technology level of TDG (Wang et al., 2015).

### Quality Evaluation Method

As is known to all, the quality of medicinal materials is closely combined with the content of active ingredients, which affects the medicinal effect of medicinal materials. TDG is a kind of Chinese herbal medicine that is commonly used among the people. On the other hand, due to its wide distribution and multiple aliases, it is easy to mix the herbs with similar appearance and similar efficacy in the application process. Therefore, the quality control evaluation standard of TDG must be established. The quality control over traditional Chinese medicine (TCM) generally includes shape identification, geographical origin identification, and determination of characteristic component contents. The methods ordinarily used for quality control of TCM include morphological, microscopic, and thinner chromatography (TLC), DNA based technologies, the chemical fingerprint analysis, etc. Yu et al. (2018) study the identification (character, microscopy, chemical reaction), inspection (moisture, total ash, heavy metals, and harmful elements), ethanol-soluble extract, and several active ingredients of TDG from Zhejiang province with the relevant methods in the China Pharmacopoeia (2015 Edition). The results of 12 batches of samples show that the total flavonoids, total polysaccharides, and total amino acids range from 0.18 to 0.66%, 21.59 to 61.76%, and 0.93 to 3.68%. And the water content, total ash content and alcohol soluble extract are 7.6~14.3%, 2.2~5.7%, and 12.4~22.6%, respectively, while the heavy metals and harmful elements such as lead, cadmium, arsenic, mercury, and copper cannot exceed the standards of the pharmacopoeia. On this basis, some researchers identify and determine acid insoluble ash, alcohol soluble extract and other inspection items for different regions of TDG, providing scientific foundation for the future development of TDG and preparations (Cui et al., 2019).

The emerging DNA-based techniques, such as inter-simple sequence repeat (ISSR), cleaved amplified polymorphic sequence (CAPS), and the internal transcribed spacer region II (ITS2) barcode have been used to distinguish TDG (Peng et al., 2016b). These methods are not suitable for the rapid identification of TDG, as they are not only complicated, time-consuming, but also expensive. In view of these disadvantages, Peng et al. (2016d) use the DNA mimic enzyme method to visually compares the true and the fake of TDG with the method of DNA mimic enzyme, to ensure the safety of clinical application of TDG. In addition, highly sensitive technologies are applied to the control the quality of traditional Chinese medicine, for example, the high-performance liquid chromatography (HPLC) with diode array detector (DAD) method (HPLC–DAD), ultra-high performance liquid chromatography tandem triple four bar mass spectrometry (UPLC-MS/MS) and infrared spectroscopy (IR), and so on. Xu et al. (2019) study the relationship between genetic and chemical diversity of TDG based on ISSR and UHPLC. They analyze the genetic differentiation of 32 wild TDG varieties with ISSR markers and determine the content of eight phenols in the leaves of these germplasms with ultra-high performance liquid chromatography (UHPLC), as well as their antioxidant activities. The results suggest that the analyzed phenolics are likely the antioxidant ingredients, and the close relationships between genetic structure and UHPLC fingerprint patterns (R^2^ = 0.9928 for genetic distance and UHPLC fingerprint). Fu C. et al. (2017) use Fourier transform near-infrared (FT-NIR) spectroscopy in combination with stoichiometric modeling techniques to quickly and effectively determine TDG and some differences, lower values, and organizational effect specifications. A kernel density estimation (KDE) based supervised discrimination method, INBC-CFSFDP is proposed. The method consists of sections: INBC is used to distinguish between known and unknown samples in the training database, and CFSFDP further distinguishes the internal classification of unknown samples. INBC and CFSFDP are combined to rapidly and effectively identify the TDG by the near-infrared spectroscopy (NIRS) from varied geographical regions. (Lin et al., 2018).

The determination to index components is a significant part of the quality control of TCM, with the determination of the content of one or several active ingredients being used as the evaluation standard for the quality control of TCM. At present, the main chemical components of TDG are flavonoids, phenolic acids, steroids, polysaccharides, and so on. With certain biological activity, all of these compounds may be used as indicators, but the related research is lack of systematicness. In this paper, we collate the relevant literature in recent years and list literature in **Table 6**. Nevertheless, it is often difficult to reflect the inherent quality of TCM as a whole with single index component, while fingerprint can reflect the quality of TCM more objectively with the characteristics of systematization, characteristic, stability, which is regarded as a quality control evaluation method of TCM. Zhang et al. (2016) establish fingerprints of chloroform parts of TDG from different origins and identify 15 peaks, of which three peaks are identified as quercetin, kaempferol-3-O-neohsperidin, and β-sitosterol. Combining principal component analysis and cluster analysis, the results show significant difference in the overall pattern of leaves and roots. Tests use ultra-high performance liquid chromatography to establish finger-prints of 41 batches of *Tetrastigma hemsleyanum* Diels et Gilg from different producing areas, and demarcate 15 common peaks in total, moreover, selecting eight major phenolic components for content determination. There is no obvious difference in the various types of chemical components, and the main difference is the content of their chemical components (Fan et al., 2016). The common mode of HPLC fingerprint of TDG decoction pieces is set up, and seven common peaks are determined, five of which are rutin, isoquercetin, kaempferol-3-o-rutoside, quercetin, and kaempferol (Zhang et al., 2018). With the expansion of the research scope, the complexity of fingerprint data has been increased, so is the difficulty of subsequent data analysis. Therefore, multivariate statistical methods have been applied to data analysis. HPLC fingerprints of TDG are collected and combined with different pattern recognition methods. Compared with principal component analysis (PCA) and partial least squares discriminant analysis (PLS-DA), random forest (RF) is a method using multiple classification trees to distinguish and classify data. Based on a mathematical model, three origin samples could be effectively distinguished in RF. Among the 18 common peaks, three of them have made outstanding contributions to different habitat sample differentiation (Li et al., 2020). The preliminary results show that this method has obvious advantages in the processing of complex data analysis, providing a technological support for the quality control and evaluation system of TDG in the future.

**Table 6:** Quantitative analysis of compounds from *Tetrastigma hemsleyanum* Diels & Gilg.

No	Extraction site	Method	Content	References
**1**	Leaves	LC−QTOF-MS LC−QqQ-MS	Qualitative analysis of chemical components and determination of Chlorogenic acid, Isoorientin, Orientin, Vitexin-2″-Orhamnoside, Vitexin, Isovitexin.	(Sun et al., 2013)
**2**	Roots	HPLC-Q-TOF-MS UPLC-QqQ-MS	Qualitative analysis of chemical components and determination of Rutin, Isoquercetin, Kaempferol-3-O-rutinoside and Astragalin.	(Xu et al., 2014a)
**3**	Roots	UPLC-MS/MS	Quantitative analysis of Procyanidin B1, Catechin, Procyanidin B2, Rutin, Isoquercitrin, Kaempferol-3-O-rutinoside, Astragalin, Quercitrin, Quercetin, and Kaempferol.	(Xu et al., 2014b)
**4**	Roots	UHPLC-DAD	Quantitative analysis of Rutin, Isoquercetin, Kaempferol-3-O-rutinoside and Astragalin.	(Fan et al., 2014)
**5**	Roots	HPLC-ELSD	Quantitative analysis of Daucosterol and β-sitosterol.	(Ding et al., 2015a)
**6**	Roots, Stem, Leaves, and Callus	HPLC and solid phase extraction technology	Quantitative analysis of Quercetin and Kaempferol.	(Liu and Qian, 2015)
**7**	Roots	UPLC-ESI-QTOF-MS/MS UPLC-QqQ-MS/MS	Qualitative analysis of chemical components and determination of Catechin, Kaempferol-3-rutinoside, Rutin, Isoquercitrin and Astragalin.	(Sun et al., 2015)
**8**	Roots	UFLC-DAD	Quantitative analysis of Procyanidins B1 and Catechin.	(Yu et al., 2016)
**9**	Leaves	UHPLC	Quantitative analysis of Neochlorogenic acid, Chlorogenic acid, Cryptochlorogenic acid, Isoorientin, Orientin, Vitexin-2-O-rhamnoside, Vitexin and Isovitexin.	(Fan et al., 2016)
**10**	Roots	HPLC	Quantitative analysis of Quercetin and β-sitosterol.	(He et al., 2017)
**11**	Leaves	RRLCQ-TOF-MS UV-Vis	Qualitative analysis of chemical components and determination of total flavonoids.	(Fan et al., 2017)
**12**	Roots, Fruits, Leaves	HPLC-DAD	Quantitative analysis of Polydatin, Isoquercitrin, Resveratrol, and Nicotiflorin.	(Zhu et al., 2017)
**13**	Leaves	UPLC-MS/MS HPLC	Qualitative analysis of chemical components and determination of Isoorientin, Orientin, Vitexin 2’’-O-rhamnoside and Vitexin, Isovitexin.	(Deng et al., 2018)
**14**	Roots	HPLC	Quantitative analysis of Rutin, Isoquercitrin, Kaempferol-3-O-rutinoside, Astragalin, Quercetin, and Kaempferol.	(Li C. et al., 2019)
**15**	Pieces	HPLC	Quantitative analysis of Rutin, Isoquercitrin, Kaempferol-3-O -rutinoside, Quercetin, and Kaempferol.	(Wang H. et al., 2019)
**16**	Roots	UPLC-Q-Exactive/MS	Qualitative analysis of chemical components and determination of Rutin, Kaempferol, Astragalin, Quercitrin, Quercetin, Vitexin-rhamnoside, Isorhamnetin, Vitexin, Emodin-8-O-β-D-glucoside, and Isoquercetin.	(Ding et al., 2019)
**17**	Leaves	RRLC-Q-TOF-MS	Qualitative analysis of chemical components and determination of Neochlorogenic acid, Chlorogenic acid, Cryptochlorogenic acid, Isoorientin, Orientin, Vitexin-2”-O-rhamnoside, Isovitexin-2”-O-rhamnoside and Orientin-2”-O- rhamnoside.	(Fang and Xu, 2017)

## Clinical Application

TDG can be used in the composition of anti-tumor prescriptions because of its remarkable capacity to anti-tumor in the clinical areas. In this respect, 120 cases of malignant tumors were treated with Jinqi tablet, which was mainly composed of TDG, Astragalus membranaceus, and Ginsenoside. It is shown that 94 cases got partial responses, and the total effective rate was 78.33% (Wei et al., 2007). Moreover, Zhonggan Mixture and Jinsidijia capsule, consisting of active pharmaceutical ingredients from TDG, have been clinically found to remarkably ameliorate the quality of daily activities of patients with advanced hepatic carcinoma and prolong life (Jiang and Gong, 2005; Chen et al., 2011). Furthermore, clinical study is carried out to find that the pathological complete response rates of 55 patients with triple negative breast cancer (abbreviated as TNBC) in the treatment group (given with a prescription containing TDG) was significantly better than that of 52 patients in the control group after different chemotherapy methods, which proved that the prescription based on TDG was effective to improve the pathological complete response rates of TNBC neoadjuvant chemotherapy (LV et al., 2014). The above-mentioned clinical practice shows that TDG has a certain therapeutic effect on tumors, so it is worthy of further clinical verification. The efficacy of clearing away heat and detoxification, eliminating stasis in TDG have been firmly general accepted in Chinese since ancient times. Such as Liu (1997) used Sanye qingyin to treat 80 children with exogenous fever, and 41 cases were cured after treatment with a total effective rate of 93.75%. Similar to the above results, 72 cases of children with exogenous fever were treated by Sanyeqing Shigao decoction, and the total effective rate reached 94.4% (Xu, 2006). Both clinical trials all gave evidence that TDG possessed more efficient on children with high fever. Finally, studies had reported that TDG had a good therapeutic effect on other diseases such as rheumatoid arthritis, mosquito bites, anal fissures and bleeding, leucorrhea, and so on. However, the well-designed clinical trials are absence, and continuous effort should be carried out for getting more reliable clinical data.

## Conclusions and Future Perspectives

This review summarizes the knowledge of TDG in phytochemistry, pharmacology, quality control, and clinical application in recent years. As the root tuber of TDG can be extremely used as a medicine, wherefore the studies become more and more. However, the focus gradually shifts to the leaves of TDG, and TDG also shares many similar pharmacological activities. Pharmacological researches show that TDG possesses includes many pharmacological effects, such as anti-tumor, anti-inflammatory, antipyretic, analgesic, and antioxidant. In addition, TDG is beneficial for lowering blood glucose and reducing blood lipids, and can also effectively treat vascular diseases. Many researchers carry out an exploration in the anti-tumor activity of TDG, briefly, mainly inhibiting tumor cell proliferation and inducing cell apoptosis through various pathways. However, the basis of anti-tumor effect of TDG is unclear. TDG is believed to have better effect of liver-protecting which is often used clinically to treat liver diseases, but the underlying mechanism of action and the relationship between chemicals and pharmacological activities should be further studied. In terms of quality control, there is lack of a complete evaluation system. Besides, the main components of content determination are flavonoids, with few studies on the content determination of other components. Furthermore, the relevant animal models had been established to verify that the clinical application of TDG is safe so that it can be taken for a long time. Simultaneously, the results of ames test, micronucleus test of bone marrow cells, and sperm deformity test of mice were negative, which proved that TDG had no mutagenicity (Jiang and Guo, 2005; Jiang and Xu, 2005). Zhong et al. (2006) had carried out an acute toxicity test, and the results showed that the maximum tolerated dose of intragastric administration in mice could reach 80 g/kg body weight, which is equivalent to 445 times of the daily clinical dosage of human beings. The above studies fully proved that TDG was safe under the clinical dose. The finding may greatly helpful to advance TDG into clinical practice. On the other hand, it had been reported that 33 compounds were found in plasma and urine after oral administration of TDG, mainly flavonoids. It speculated that the main metabolic reactions may be glucuronidation, sulfation, deglycosylation, and methylation in the body (Sun et al., 2015). However, there were few researches about pharmacokinetics so that we need more to understand the complex components of TDG’s metabolism in the body, and clarified the effective ingredients, which was conducive to improving the clinical application value of TDG.

Finally, researches in the future should be developed through some new technologies, such as proteomics, metabolomics, analytical methods, and so on, to clarify the potential mechanism of TDG pharmacological activity and promote the clinical therapeutics. Few studies have been conducted on the adverse effects or toxicity of different extracts and its active ingredients, so a basic research on this aspect should be taken into consideration. Mostly, research on pharmacological effects has focused on the cellular level, so appropriate animal models are required for more *in vivo* studies. The studies in the future should place more emphasis on systematically investigating the pharmacological mechanism of TDG, as well as the establishment of a comprehensive quality standard of TDG, so as to better promote the development and utilization of the medicinal value and clinical application of TDG.

## Author Contributions

RZ collated documents and wrote the manuscript. XW and GC contributed significantly to the outline and revised the manuscript. XX and JY helped with summarizing the table on compatibility application.

## Funding

This study was supported by the National Natural Science Foundation of China (No.81803840).

## Conflict of Interest

The authors declare that the research was conducted in the absence of any commercial or financial relationships that could be construed as a potential conflict of interest.
